# The Active Tamoxifen Metabolite Endoxifen (4OHNDtam) Strongly Down-Regulates Cytokeratin 6 (CK6) in MCF-7 Breast Cancer Cells

**DOI:** 10.1371/journal.pone.0122339

**Published:** 2015-04-13

**Authors:** Thomas Helland, Jennifer Gjerde, Simon Dankel, Ingvild S. Fenne, Linn Skartveit, Andreas Drangevåg, Olivera Bozickovic, Marianne Hauglid Flågeng, Håvard Søiland, Gunnar Mellgren, Ernst A. Lien

**Affiliations:** 1 Hormone Laboratory, Haukeland University Hospital, Bergen, Norway; 2 Department of Clinical Science, University of Bergen, Bergen, Norway; 3 Section for Breast and Endocrine Surgery, Stavanger University Hospital, Stavanger, Norway; Roswell Park Cancer Institute, UNITED STATES

## Abstract

**Introduction:**

Tamoxifen is an anti-estrogen drug used in treatment of Estrogen Receptor (ER) positive breast cancer. Effects and side effects of tamoxifen is the sum of tamoxifen and all its metabolites. 4-Hydroxytamoxifen (4OHtam) and 4-hydroxy-*N*-demethyltamoxifen (4OHNDtam, endoxifen) both have ER affinity exceeding that of the parent drug tamoxifen. 4OHNDtam is considered the main active metabolite of tamoxifen. Ndesmethyltamoxifen (NDtam) is the major tamoxifen metabolite. It has low affinity to the ER and is not believed to influence tumor growth. However, NDtam might mediate adverse effects of tamoxifen treatment. In this study we investigated the gene regulatory effects of the three metabolites of tamoxifen in MCF-7 breast cancer cells.

**Material and Methods:**

Using concentrations that mimic the clinical situation we examined effects of 4OHtam, 4OHNDtam and NDtam on global gene expression in 17β-estradiol (E_2_) treated MCF-7 cells. Transcriptomic responses were assessed by correspondence analysis, differential expression, gene ontology analysis and quantitative real time PCR (Q-rt-PCR). E_2_ deprivation and knockdown of Steroid Receptor Coactivator-3 (SRC-3)/Amplified in Breast Cancer 1 (AIB1) mRNA in MCF-7 cells were performed to further characterize specific effects on gene expression.

**Results:**

4OHNDtam and 4OHtam caused major changes in gene expression compared to treatment with E_2_ alone, with a stronger effect of 4OHNDtam. NDtam had nearly no effect on the global gene expression profile. Treatment of MCF-7 cells with 4OHNDtam led to a strong down-regulation of the CytoKeratin 6 isoforms (*KRT6A*, *KRT6B* and *KRT6C*). The CytoKeratin 6 mRNAs were also down-regulated in MCF-7 cells after E_2_ deprivation and after SRC-3/AIB1 knockdown.

**Conclusion:**

Using concentrations that mimic the clinical situation we report global gene expression changes that were most pronounced with 4OHNDtam and minimal with NDtam. Genes encoding CytoKeratin 6, were highly down-regulated by 4OHNDtam, as well as after E_2_ deprivation and knockdown of SRC-3/AIB1, indicating an estrogen receptor-dependent regulation.

## Introduction

The Selective Estrogen Receptor Modulator (SERM) tamoxifen is used in breast cancer treatment and prevention. It may act as a full estrogen agonist, partial agonist or antagonist depending on the dose, species, or target organ [1]. Tamoxifen is regarded as a pro-drug since two of its metabolites, 4-hydroxytamoxifen (4OHtam) and 4-hydroxy-*N*-demethyltamoxifen (4OHNDtam, endoxifen), both have Estrogen Receptor (ER) affinity markedly exceeding that of tamoxifen itself [2–4]. 4OHNDtam is considered the main active metabolite of tamoxifen since it has 100-fold higher affinity for the ER than tamoxifen and its serum levels are 10-fold higher than that of 4OHtam [5–9]. During steady state tamoxifen treatment the concentrations of 4OHtam, 4OHNDtam and Ndesmethyltamoxifen (NDtam) are roughly present in serum in concentrations 5, 50 and 150% respectively compared to that of tamoxifen [10–12]. However in the clinical situation, these concentrations vary up to tenfold between patients using an identical daily dose, furthermore the concentrations increase by increasing age [9, 13].

Properties of tamoxifen metabolites may be studied in the ER positive human breast cancer cell line MCF-7. The majority of *in vitro* studies on effects of tamoxifen are using 4OHtam as single drug, whereas studies including 4OHNDtam as single drug are used only in few *in vitro* studies. Lim *et al* observed that 4OHNDtam and 4OHtam have similar effects on the global expression pattern in MCF-7 cells, especially on the estrogen-regulated genes [14]. Hawse *et al* also studying global gene expression in MCF-7 cells observed that 4OHNDtam molecular mechanism of action was concentration dependent and different than that of other anti-estrogens [15]. High but not low concentrations of 4OHNDtam resulted in induction of cell cycle arrest and markers of apoptosis [15]. Recently, effects of 4OHNDtam as single drug have been examined in animal studies [16, 17] and at present clinical studies using 4OHNDtam as single drug are underway [15]. NDtam is the major tamoxifen metabolite in serum, but due to a low affinity to the ER NDtam is believed not to influence tumor growth and little attention has been drawn to the compound.

The effects and side effects represent a summary of effects of tamoxifen and all its metabolites. In two earlier studies where the hydroxylated metabolites were not measured, it was observed that the proportion of tamoxifen and its demethylated metabolites NDtam and N-desdimethyltamoxifen (NDDtam) in serum was higher in patients with toxicity versus those not experiencing toxicity [18, 19]. More recent studies report that women with higher 4OHNDtam levels are more likely to report side effects [13, 20].

In the present explorative study we examined effects of the major demethylated tamoxifen metabolite NDtam and the hydroxylated potent metabolites 4OHtam and 4OHNDtam on global gene expression in MCF-7 cells using concentrations that are representative for the clinical situation [9, 21]. We also studied differences in effects between 4OHtam and 4OHNDtam which may influence tumor growth and searched for genes that had the most extensive changes in gene expression profile.

## Materials and Methods

### Cell culture

Michigan Cancer Foundation-7 (MCF-7) human breast adenocarcinoma cells [22] were grown at 37°C under 5% CO2, in DMEM (Invitrogen, Carlsbad, CA, USA) supplemented with 10% fetal bovine serum, 1% (vol/vol) penicillin/streptomycin solution and 4.5 g/liter glucose. The MCF-7 cell medium also contained 1 μM insulin. The MCF-7 cell line is a commercial cell line in which no ethical approval is required for experiments.

### Cell treatments

#### Treatment of MCF-7 cells with tamoxifen metabolites

The cells were preconditioned in phenol red-free DMEM (Invitrogen, Carlsbad, CA) containing charcoal-stripped fetal bovine serum (Hyclone^TM^, Thermo Fischer Scientific, MA, USA) and the above supplements, for 2 days. The cells were seeded in six-well plates at a density of 300,000 cells/ml and then treated with 10nM E_2_ alone or in combination with 4OHNDtam, 4OHtam or NDtam (Table 1) for three days. E_2_ and 4OHtam (>70% Z isomer) were purchased from Sigma-Aldrich (Steinheim, Germany) and 4OHNDtam (Z/E isomers 1/1) from Sintef Materials and Chemistry (Oslo, Norway). NDtam was a gift from Imperial Chemical Industries, PLC Pharmaceutical divisions (Macclesfield, UK). Cells were harvested after 3 days of incubation for the microarray analysis. The growth medium from the incubated cell cultures was collected and the concentrations of tamoxifen and its metabolites determined by High Pressure Liquid Chromatography (HPLC)—Tandem Mass Spectrometry (MS/MS) [8, 23].

**Table 1:** Concentrations (ng/ml) of tamoxifen and its metabolites in growth media.

	**NDtam**	**4OHtam**	**4OHNDtam**
	**Concentration (ng/ml) **		
**Calculated**			
Day 0	1000	100	1000
**Measured**			
Day 1	939	72,6	609
Day 2	158	16	64,9
Day 3	119 (93–133)	6.0 (4.0–7.7)	36.6 (26.4–54.4)

Concentrations determined by High Pressure Liquid Chromatography (HPLC)—Tandem Mass Spectrometry (MS/MS). Three measurements performed at day 3. One measurement performed at day 1 and 2.

#### Estrogen deprivation and knockdown of SRC-3 in MCF-7 cells

MCF-7 cells were grown in alpha MEM (Lonza, Belgium) supplemented with 10% fetal bovine serum (FBS), 2 mM L-glutamine, 100 units penicillin, 100 μg streptomycin and 1 μM insulin for one day and then grown in phenol red-free Alpha MEM supplemented with 5% charcoal-stripped FBS for 3 days. MCF-7 cells grown for 3 days with the addition of 10 nM 17β-estradiol (Sigma) were used as control.

Short Hairpin RNA (shRNA) lentiviral transduction was used to generate MCF-7 cells containing stably integrated shRNA SRC-3/AIB1 mRNA as previously described [24]. 68% reduction in SRC-3/AIB1 mRNA expression was obtained after KD of SRC-3 (SRC-3 shRNA) compared to the control shRNA.

### Homogenization and RNA extraction

Lysates from cell samples were harvested in PBS and RNA was extracted using the RNeasy Mini Kit (Qiagen, Hilden, Germany) according to the manufacturer’s recommendations. Samples were treated with the RNase-Free DNase Set (Qiagen). Amount and quality of the extracted RNA were measured by the NanoDrop ND-1000 spectrophotometer (NanoDrop Technologies, ThermoScientific, Waltham, MA, USA) and the Agilent 2100 Bioanalyzer (Agilent Technologies, Santa Clara, CA, USA).

### Illumina iScan system

250 ng of RNA from the cell samples treated with E_2_, NDtam, 4OHtam and 4OHNDtam were biotin-labeled and amplified using the Illumina TotalPrep RNA amplification kit (Ambion, Austin, TX, USA) and the Eppendorf Mastercycler (Eppendorf Hamburg, Germany). This procedure involves RNA being reversely transcribed, amplified and biotin-16-UTP-labeled. The biotin-labeled cRNA was thereafter quality and quantity controlled using the Agilent 2100 Bioanalyzer and the NanoDrop ND-1000 spectrophotometer. 1500 ng of cRNA was hybridized to the humanWG-6 v.3.0 expression BeadChip (Illumina, San Diego, CA, USA) and the fluorescence of the biotin-labeled cRNA was detected using the Illumina iScan.

### Microarray data extraction and analysis

#### Quality control and preprocessing

After scanning in the iScan reader the microarray raw data was imported into GenomeStudio software (Illumina) which removed control probes and produced a text file that contained the signal and detection p-values per probe for all samples. This text file was further imported into J-Express 2009 (MolMine AS, Norway) where signal intensity values were quantile normalized and logarithmically transformed (base 2) [25]. Quantile normalization removes obscuring variations that arise from differences in the preparation of the microarray samples. A Correspondence Analysis (CA) and hierarchical clustering with Pearson Correlation as a distance measure were performed to visualize the differential expression between the four differently treated groups and analyze global trends in the data [26].

In adherence to the standards of the Microarray Gene Expression Data Society (mged) the microarray data is publicly available at ArrayExpress under the title “Tamoxifen treatment of MCF-7 breast cancer cells” and accession number (E-MTAB-2729).

#### Analyses of differentially expressed genes

To search for differentially expressed genes in MCF-7 breast cancer cells treated with E_2_, NDtam, 4OHtam or 4OHNDtam a Significance Analysis of Microarrays (SAM) [27] was applied. The SAM analysis calculates the significance of the gene expression based on the deviation between the actual signal intensity and the signal intensity expected by chance. To obtain manageable datasets, differentially expressed genes were defined by q-value = 0. A rank product analysis was set up to examine if there were genes that were more regulated by one metabolite than the other. By using this non-parametric statistical method it was possible to rank the genes according to fold change and compare several rank product lists against each other. The analysis was performed using J-express (Molmine) and Excel (Microsoft).

To analyze which Gene Ontology (GO) functional groups the differentially expressed genes belonged to Protein ANalysis THrough Evolutionary Relationships (PANHTER) (dated, 15. February, 2012) [28] was applied. PANTHER identifies which functional groups are over-represented among a selection of genes and expresses the degree of over-representation with p-values (binomal statistics). The integrated gene ontology analysis in J-Express 2012 (Molmline) software was used to validate the findings from PANTHER. Gene lists used for PANTHER were compared to the entire gene list from the microarray and an over-representation analysis was performed.

### Quantitative-real time-PCR

1 μg or 350 ng RNA per reaction was transcribed to cDNA using the Transcriptor First Strand cDNA Synthesis Kit (Roche Diagnostics, GmbH, Mannheim, Germany) and further quantified using the LightCycler480 Probes Master kit (Roche) and the LightCycler480 rapid thermal cycler system (Roche). Each gene was quantified relative to reference genes TATA-binding protein (TBP), glucose-6-phosphate-1-dehydrogenase (G6PD) or peptidylprolyl isomerase A / Cyclophilin A (PPIA) to correct for variations occurring within samples and during sample preparations. The quantification of the genes was performed using specific Universal ProbeLibrary (UPL) probes and target-specific primers, designed at Universal ProbeLibrary (UPL) Assay Design Center (Roche, Applied Science), software version 2.45 (S1 Table). The relative quantification of the genes was performed according to manufactures protocol (Roche Applied Science).

## Results

**Fig 1:**
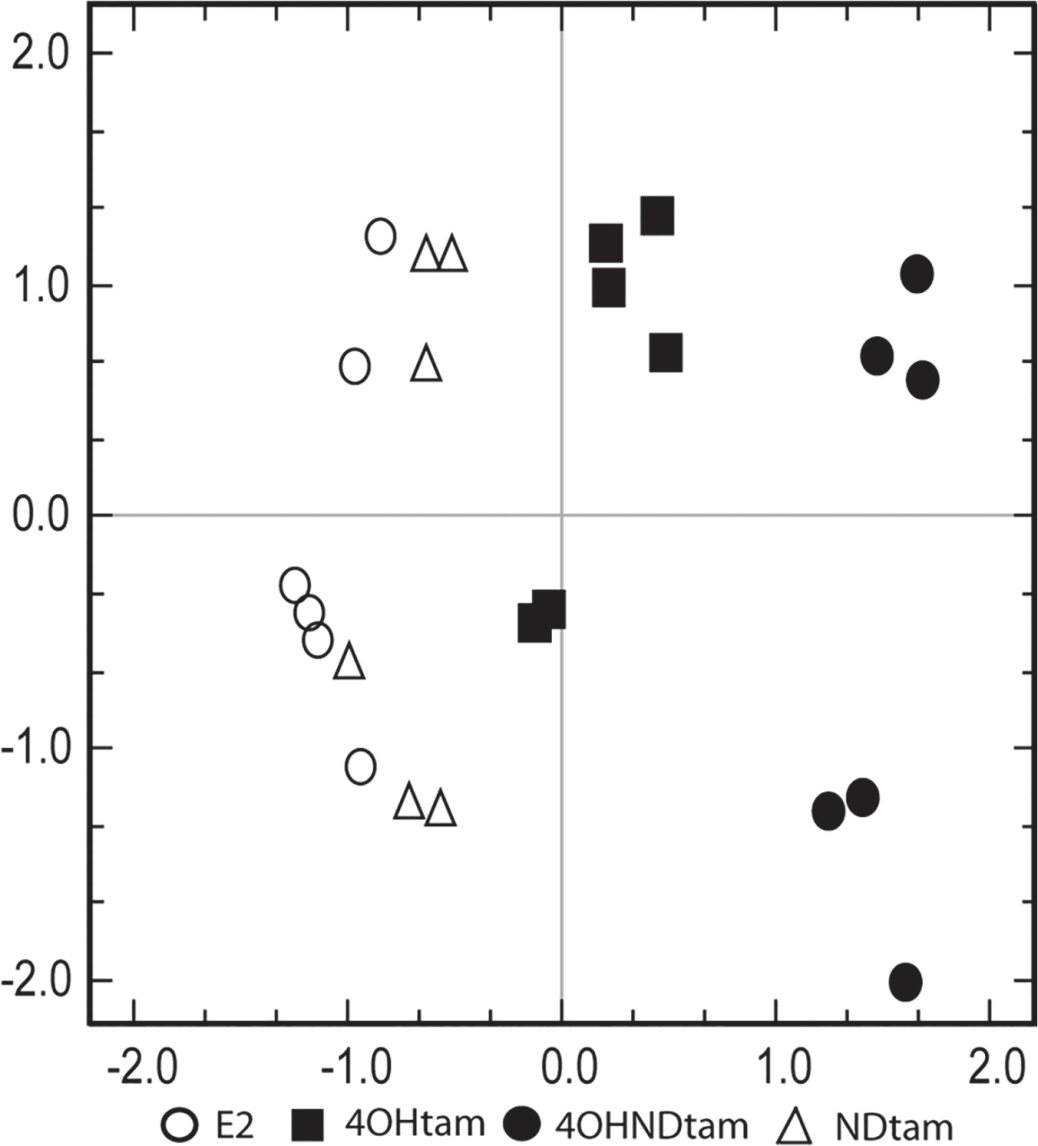
Correspondence analysis showing projection of MCF-7 cell samples treated with E_2_, NDtam, 4OHtam and 4OHNDtam. The x-axis displays the first principle component (variance: 8.01%) and y-axis displays the second principle component (variance: 6.959%). The MCF-7 cell samples; MCF-7 treated with only E_2_ (blank circle), MCF-7 treated with E_2_ and NDtam (blank triangle), MCF-7 treated with E_2_ and 4OHtam (black square), and MCF-7 treated with E_2_ and 4OHNDtam (black circle).

To elucidate the gene regulative roles of the three tamoxifen metabolites 4OHtam, 4OHNDtam and NDtam in breast cancer, we performed a microarray analysis on MCF-7 cells. The MCF-7 cells were treated with the respective tamoxifen metabolites and E_2_ for three days with concentrations intended to mimic physiological concentrations (Table 1). We used the concentration levels found in tissues which are approximately 10 times higher than the concentrations found in serum [29]. Although the inter-individual serum concentrations of tamoxifen and its main metabolites vary tenfold and increase by age [9], the ratio between 4OHNDtam/4OHtam is usually 10/1 in the individual patient [9, 21]. Therefore, we used a concentration of 4OHNDtam ten times higher than that of 4OHtam. To analyze the differential expression on a global level between the four compounds (E_2_, 4OHtam, NDtam and 4OHNDtam), a Correspondence Analysis (CA) was performed (Fig 1). The CA, displaying global gene expression in a two-dimensional plot, showed that the samples treated with 4OHtam and 4OHNDtam were clearly separated from control (E_2_). 4OHtam and 4OHNDtam shifted gene expression in the same direction, but the shift was greater for 4OHNDtam (Fig 1). The samples treated with NDtam were clustered closely with the E_2_ control suggesting limited effect on gene expression in MCF-7 cells.

**Fig 2:**
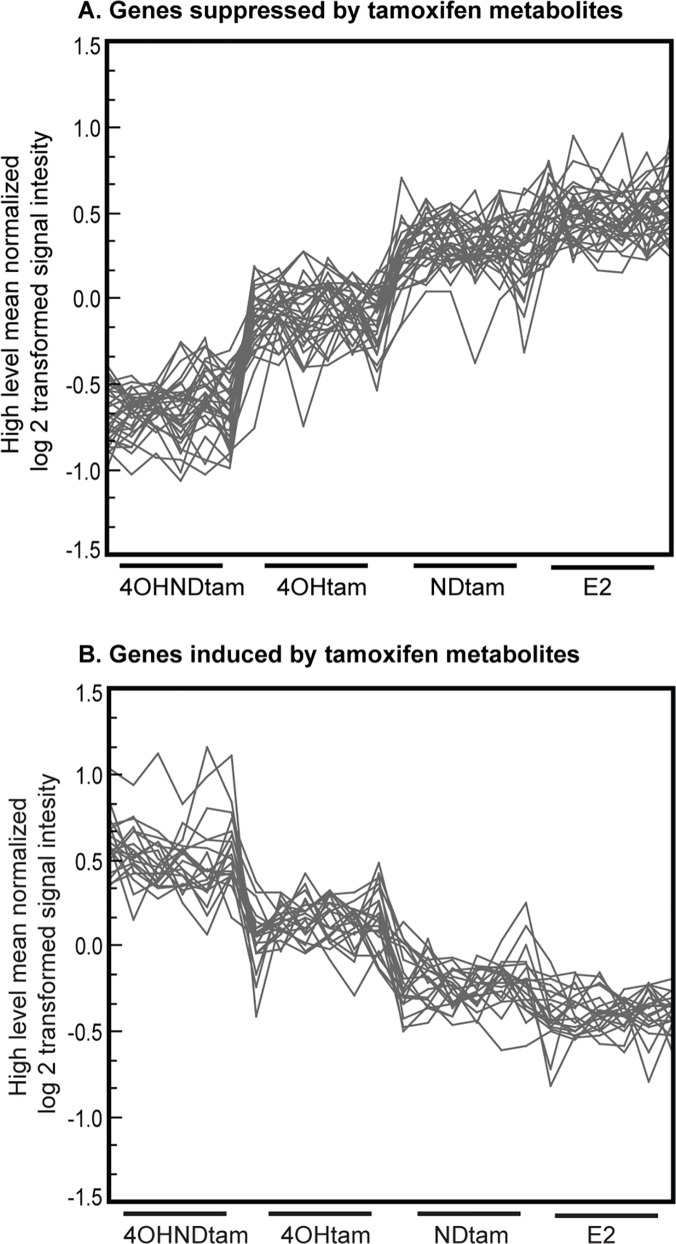
Self-organizing maps displaying stepwise regulation between 4OHNDtam and 4OHtam. Self-organizing map displaying the high level mean normalized log2 transformed signal intensities (y-axis) across different treatments (x-axis). Each treatment has 5 parallel samples (x-axis). Figure A displays a clustering of down-regulated genes in a stepwise regulation where 4OHNDtam is the strongest down-regulated. Figure B shows a clustering of up-regulated genes regulated in a stepwise regulation with 4OHNDtam being the most up-regulated.

To further visualize gene expression trends in our dataset we performed a self-organizing map analysis. This clustering analysis was performed on high level mean normalized expression values for genes with a significant expression value (q-value = 0) when comparing treatment to control (E_2_). The results showed a stepwise regulation between 4OHtam and 4OHNDtam, where 4OHNDtam resulted in the most differential expression (Fig 2).

**Fig 3:**
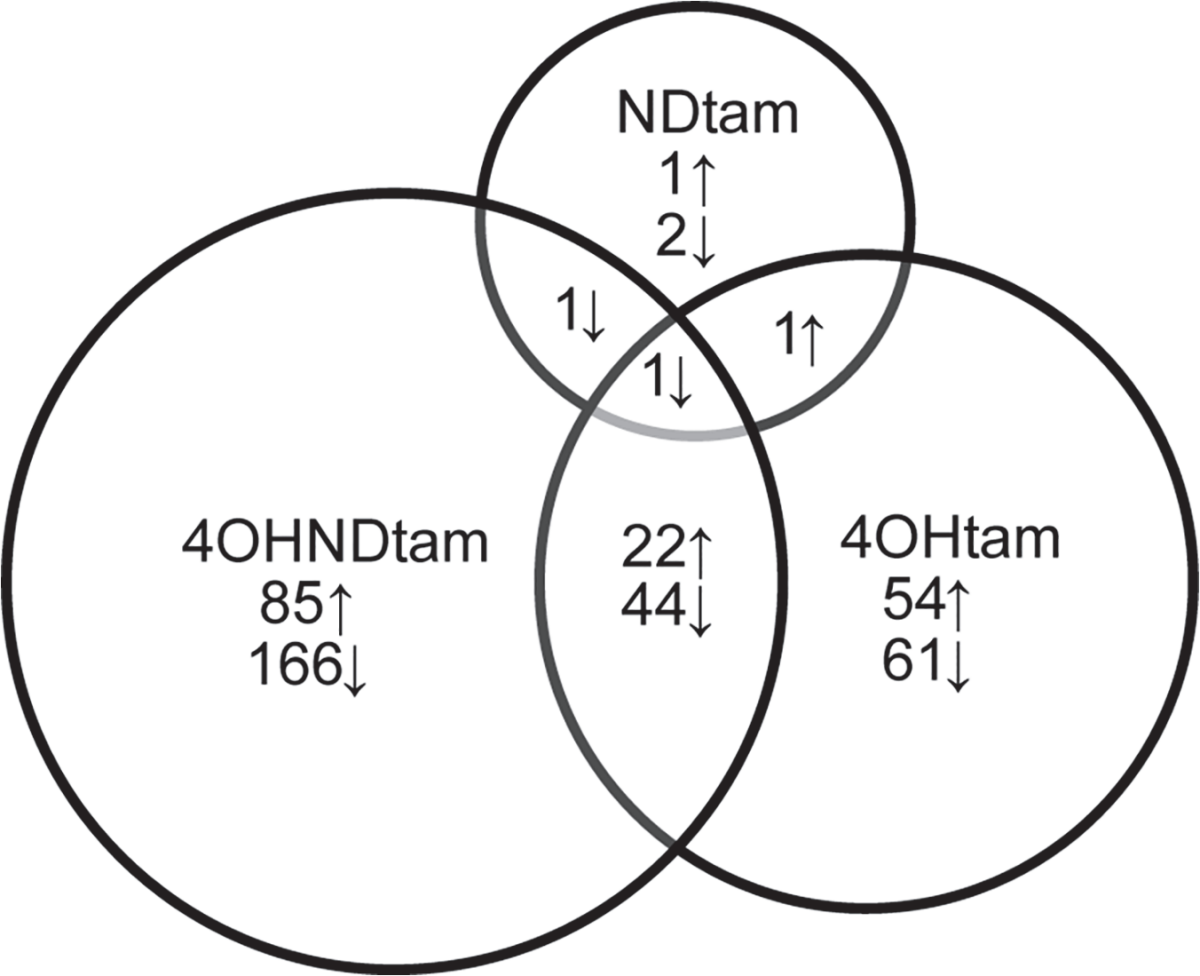
Venn diagram showing genes uniquely regulated and regulated in the same direction after treatment with tamoxifen metabolites. Genes included have a fold change ≥ |1.5| and q-value = 0 when comparing tamoxifen metabolites to E_2_. The numbers in the overlapping circles shows the number of genes regulated in the same direction by different treatments.

A Significance Analysis of Microarray (SAM) identified the differentially expressed genes for each metabolite compared to control (E_2_). Only six NDtam-regulated genes met the 1.5-fold change and q-value = 0 cut-off for differential expression, but half of these genes overlapped with one or both of the other treatment groups (Fig 3). Of the 251 genes regulated by 4OHNDtam and 115 regulated by 4OHtam, there were 66 overlapping genes, i.e. 57% of the 4OHtam-regulated genes were also regulated in the same direction at least 1.5-fold by 4OHNDtam. Differential expression observed in the microarray (S2–S5 Tables) was confirmed using Q-rt-PCR on a selection of genes with high differential expression (Table 2).

**Table 2:** Validation of microarray fold change values using Q-rt-PCR.

**Target gene**		**NDtam**	**4OHtam**	**4OHNDtam**
KRT6A	Microarray	0.99	0.93	0.73
	Q-rt-PCR	0.84 (0.52–1.07)	0.49 (0.23–1.06)	0.06 (0.05–0.08)
KRT6C	Microarray	0.99	0.94	0.78
	Q-rt-PCR	1.08 (0.7–1.32)	0.69 (0.24–1.40)	0.11 (0.05–0.12)
*CXCR4 tv2*	Microarray	0.9	0.694	0.63
	Q-rt-PCR	0.68 (0.51–0.90)	0.45 (0.37–0.54)	0.35 (0.27–0.47)
*SERPINA3*	Microarray	0.73	0.3	0.12
	Q-rt-PCR	0.64 (0.58–0.69)	0.21 (0.18–0.24)	0.07 (0.05–0.08)
*GPER TV4*	Microarray	0.92	0.39	0.27
	Q-rt-PCR	0.85 (0.76–0.94)	0.34 (0.26–0.43)	0.24 (0.15–0.35)
*CTGF*	Microarray	1.14	1.1	2.24
	Q-rt-PCR	1.05 (0.82–1.28)	1.54 (1.26–1.83)	1.15 (0.97–1.32)
*COL3A1*	Microarray	1.49	2.427	2.56
	Q-rt-PCR	2.03 (1.36–2.70)	5.86 (3.89–7.81)	6.11 (3.46–8.77)
*IRX2*	Microarray	0.91	0.64	0.43
	Q-rt-PCR	0.88 (0.80–0.97)	0.48 (0.44–0.52)	0.47 (0.42–0.53)
*IRX3*	Microarray	0.95	0.59	0.55
	Q-rt-PCR	0.85 (0.75–0.97)	0.43 (0.38–0.48)	0.42 (0.35–0.52)
*IRX5*	Microarray	0.97	0.64	0.57
	Q-rt-PCR	0.94 (0.92–0.97)	0.55 (0.52–0.58)	0.59(0.55–0.64)

Median values of fold change (E_2_/treatment). Numbers in brackets are min-max values. Fold changes calculated from signal intensity (microarray) and concentration (Q-rt-PCR). N = 6 for each treatment.

**Fig 4:**
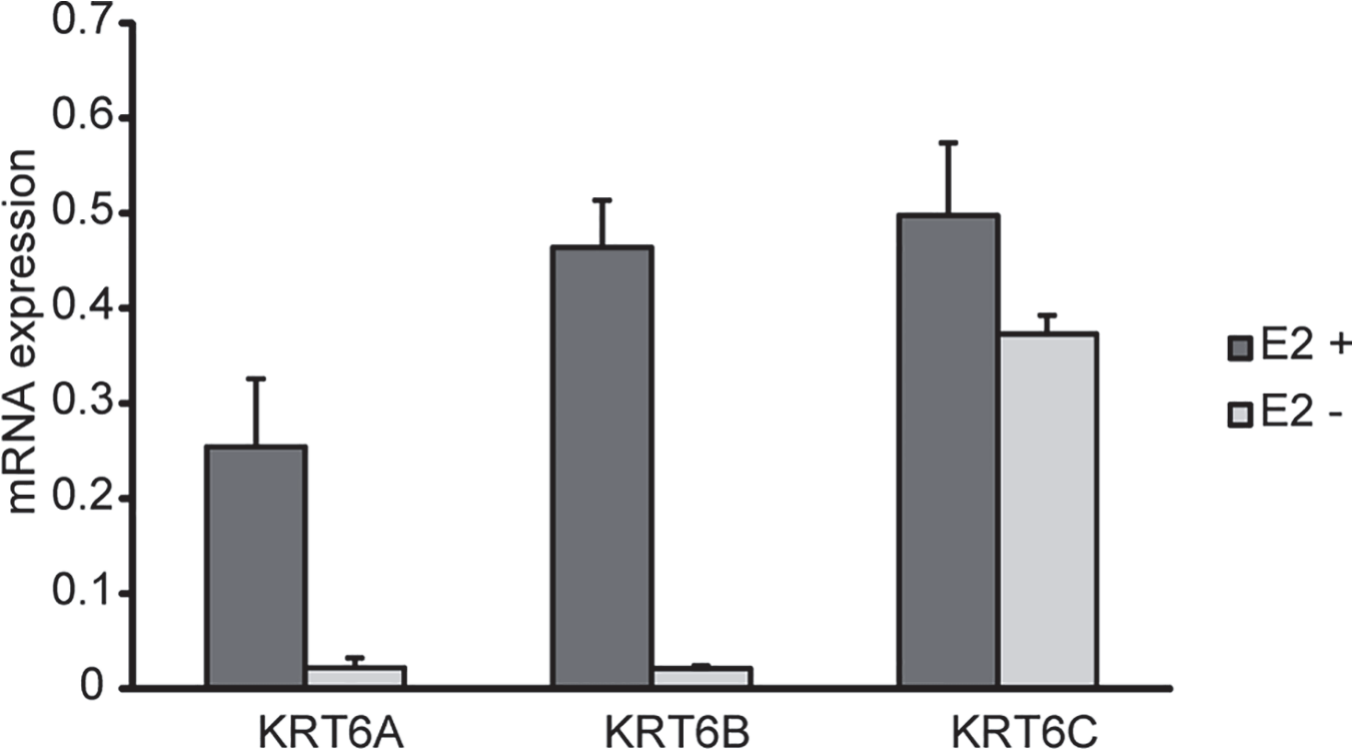
KRT6 mRNA expression after estrogen deprivation in MCF-7 cells. MCF-7 cells were grown in phenol red-free Alpha MEM supplemented with 5% charcoal-stripped FBS for 3 days. Cells grown for 3 days in presence of 17β-estradiol (10 nM) were used as control. The mRNA expression was measured by Q-rt-PCR and the relative expression levels of each gene were related to TBP mRNA. The results presented are mean values with SEM from 3 biological replicates.

**Fig 5:**
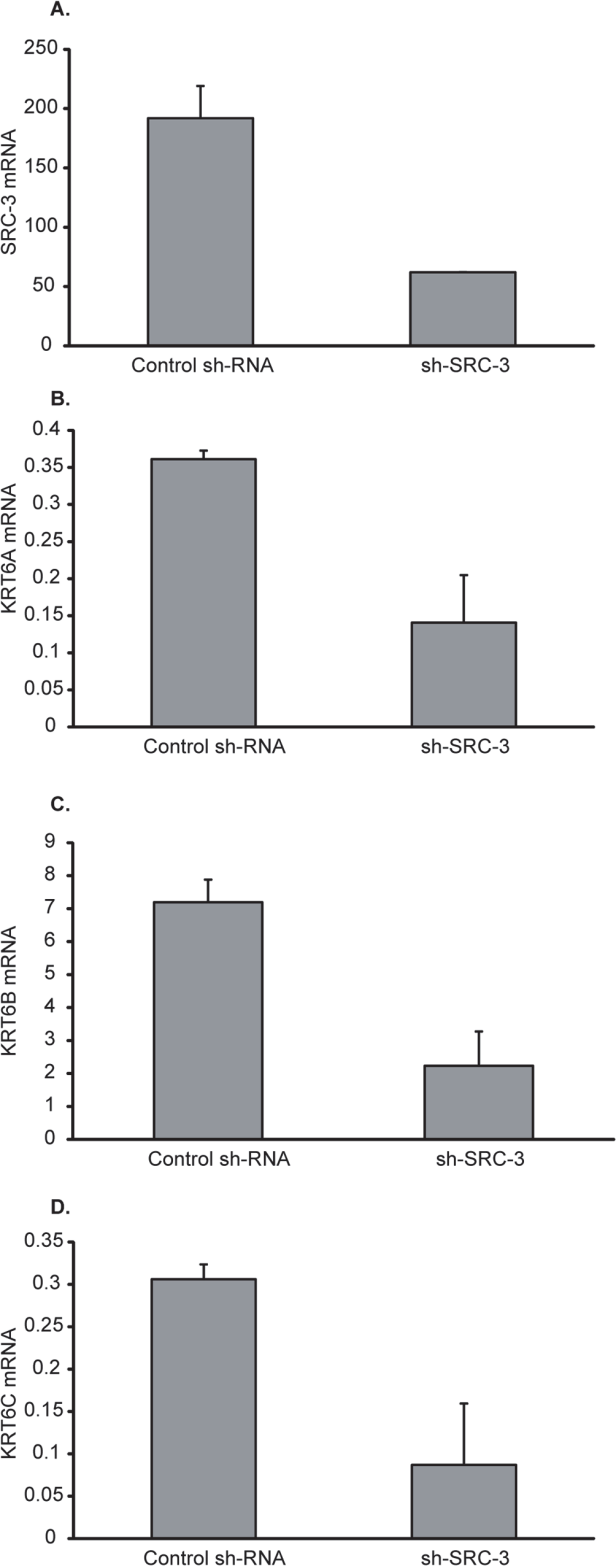
KRT6 mRNA expression after knockdown of SRC-3 in MCF-7 cells. **A:** Quantification of SRC-3 mRNA expression in MCF-7 cells infected with shRNA targeting SRC-3 (SRC-3 shRNA) and Control shRNA. **B-D:** mRNA expression of KRT6A, KRT6B and KRT6C in SRC-3 shRNA- and Control shRNA MCF-7 cells. The expression level of each gene is relative to TBP mRNA. The results presented are mean values with SEM from 6 biological replicates.

In the gene list for 4OHNDtam treated cells, the cytokeratin 6 (KRT6) genes were strikingly down-regulated compared to control (E_2_). *KRT6A* was down-regulated 9.1-fold compared to control while *KRT6B* and *KRT6C* were down-regulated 3.3- and 4.1-fold, respectively (S3 Table). The strong down-regulation of these genes was confirmed by Q-rt-PCR (Table 2). To further explore whether the down-regulation of the KRT6s were regulated through estrogen receptor signaling we did two separate Q-rt-PCR analyses. Firstly, we cultured the cells in absence of E_2_ for 3 days and examined the expression levels of the three KRT6s by Q-rt-PCR (Fig 4). Estrogen deprivation of the MCF-7 cells for 3 days nearly abolished the mRNA expression of KRT6A and B. Estrogen deprivation also down-regulated the KRT6C mRNA levels, but to a less extent than KRT6A and B. Secondly, we knocked down the ligand-dependent ER-coactivator Steroid Receptor Coactivator 3 (SRC-3), also known as Amplified in Breast Cancer 1 (AIB1) (Fig 5). SRC-3/AIB1 knockdown also repressed the expression of all three KRT6s. Based on these data, we conclude that the KRT6s are positively regulated by E_2_.

**Fig 6:**
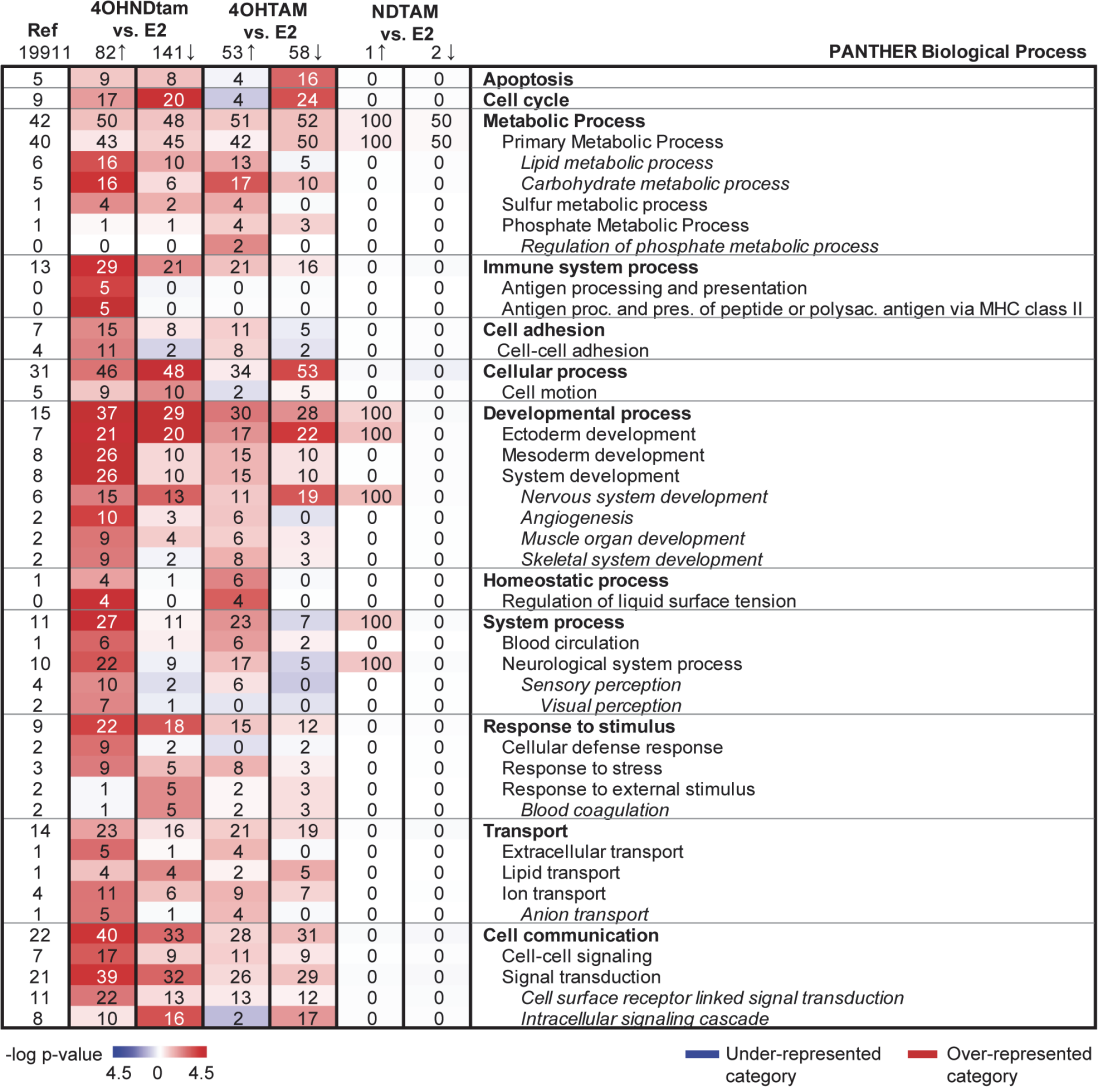
Functional categorization of differentially expressed genes in MCF-7 cells after treatment with tamoxifen metabolites. PANTHER was used to search for over-represented categories in the ontology class Biological Process. A significance of microarray analysis (SAM) was applied to search for genes that were differentially expressed after treatment with the three different tamoxifen metabolites. Genes with q-val ≤ 0 and fold change ≥1.5 were selected from the SAM analysis. A p-value ≤ 0.05 was used as inclusion criterion for categories. The reference column at the left of the table displays the percentage of genes that belongs to a specific category when analyzing the whole human NCBI genome (19,911 genes), e.g. 5% of the 19,911 genes belong to the “Apoptosis” category. The same principle goes for the other numerical columns, e.g. in the 4OHNDtam vs. E_2_ column there are 82 up-regulated genes and 9% of these belonged to the “Apoptosis” category. The color intensity scales are based on the statistical significance (-log p-value) of over- and under-represented PANTHER functional categories. Red illustrates “over-represented category” where more genes than expected were found in a specific category. Blue color illustrates “under-represented category” where less genes than expected were found. Ref, Reference genes. Arrow up, up-regulated genes. Arrow down, down-regulated genes.

To inform on functions of the differentially expressed genes after treatment with the three different metabolites (fold change ≥ 1.5, q-val = 0) we performed the Gene Ontology (GO) analysis with PANTHER. Differentially expressed genes after treatment with 4OHNDtam and 4OHtam were largely enriched in the same GO categories (Fig 6). However, the categories are more over-represented after treatment with 4OHNDtam compared to 4OHtam. This may reflect the fact that there are more genes that met the cut of criteria for the clinical applicable concentration (1000 ng/mL) of 4OHNDtam (82↑, 142↓) in contrast to the lower concentration (100 ng/mL) of 4OHtam (53↑, 58↓). NDtam on the other hand, has no representation in PANTHER because the fold change of 1.5 only resulted in 3 differentially expressed genes despite having a concentration of 1000 ng/mL. Studying specific categories in PANTHER we observed genes belonging to the category cell cycle to be down-regulated after both treatments (4OHtam and 4OHNDtam), while genes involved in cell adhesion were up-regulated. In addition, treatment of MCF-7 cells with 4OHtam resulted in a clear down-regulation of genes involved in apoptosis, while treatment with 4OHNDtam resulted in both up- and down-regulation of the apoptosis related genes. Of particular note, only 4OHNDtam showed a significant effect on genes related to antigen processing and presentation (up-regulation). To validate our findings in the PANTHER analysis we performed an alternative GO over-representation analysis using the integrated GO analysis software in J-Express 2012. This analysis resulted in predominantly the same over-represented categories as in PANTHER (S6 Table).

To further analyze the metabolite-specific potency to regulate gene expression, we searched for genes that were differentially expressed after treatment with 4OHtam and at the same time more differentially expressed after treatment with 4OHNDtam (rank product analysis, fold change ≥1.5 for each step and q-value ≤ 0.2). These cut-off values resulted in 20 up-regulated genes and 81 down-regulated genes, that we subjected to PANTHER analysis to evaluate their function (S1 Fig). We found that the categories developmental processes and signaling, including G-protein coupled receptor protein signaling pathway, were over-represented among the down-regulated genes (fold change ≤ 0.05, S7 Table). Genes involved in neurological processes such as sensory perception were found to be over-represented among the up-regulated genes (S8 Table). The cancer related category apoptosis was over-represented with both up- and down-regulated genes, represented by two up-regulated genes (*TNS3*, *EMP1*) and five genes down-regulated (*DNASE1L2*, *CXCR4*, *Gal-7*, *FKBP5*, *SGK1*) (Table 3).

**Table 3:** Genes with differential expression after treatment with 4OHtam and more differential expression after treatment with 4OHNDtam belonging to biological process category (PANTHER).

	**Up-regulated**	**Down-regulated**
**Developmental process**	*VGLL1*, *BASP1*, *EMP1*, *KRT4*	*KRT6A*, *CXCR4*, *TUBA3D*, *EGR3*, *EGR2*, *MGP*, *KRT17*, *PRLR*, *IRX2*, *HEY2*, *SIAH2*, *OLFM1*, *KRT6C*, *IL27RA*, *TUBA3D*, *KRT6B*
System development	*BASP1*	*CXCR4*, *EGR3*, *EGR2*, *PRLR*, *MGP*, *IRX2*, *HEY2*, *SIAH2*, *OLFM1*, *IL27RA*
*Nervous system development*	*BASP1*	*CXCR4*, *EGR3*, *EGR2*, *IRX2*, *HEY2*, *SIAH2*, *OLFM1*
Ectoderm development	*BASP1*, *EMP1*, *KRT4*	*KRT6A*, *CXCR4*, *EGR3*, *EGR2*, *IRX2*, *HEY2*, *SIAH2*, *OLFM1*, *KRT6C*, *KRT6B*
**Cellular process**	*TNS3*, *EMP1*, *KRT4*, *INHBB*, *CAV1*, *GABRP*	*KRT6A*, *GPR68*, *RERG*, *CXCR4*, *TUBA3D*, *TGFA*, *CCBP2*, *Gal-7*, *FXYD4*, *FKBP5*, *EGR3*, *EGR2*, *GPER*, *MGP*, *RAB31*, *KRT17*, *PRLR*, *CCBP2*, *SGK1*, *Gal-7*, *GEM*, *PPP2R5A*, *ANXA8*, *OLFM1*, *KRT6C*, *IL27RA*, *TUBA3D*, *KRT6B*, *OXTR*, *PKIB*
Cell communication	*TNS3*, *INHBB*, *CAV1*, *GABRP*	*GPR68*, *RERG*, *CXCR4*, *TGFA*, *CCBP2*, *FXYD4*, *GPER*, *FKBP5*, *MGP*, *RAB31*, *PRLR*, *SGK1*, *GEM*, *CCBP2*, *PPP2R5A*, *ANXA8*, *OLFM1*, *IL27RA*, *OXTR*, *PKIB*
*Signal transduction*	*TNS3*, *INHBB*, *CAV1*, *GABRP*	*GPR68*, *RERG*, *CXCR4*, *TGFA*, *CCBP2*, *FXYD4*, *GPER*, *FKBP5*, *MGP*, *RAB31*, *PRLR*, *SGK1*, *GEM*, *CCBP2*, *PPP2R5A*, *ANXA8*, *OLFM1*, *IL27RA*, *OXTR*, *PKIB*
*Intracellular signaling cascade*		*RERG*, *RAB31*, *PRLR*, *FKBP5*, *SGK1*, *GEM*, *PPP2R5A*, *IL27RA*, *OXTR*, *PKIB*
*Apoptosis*	*TNS3*, *EMP1*	*DNASE1L2*, *CXCR4*, *Gal-7*, *FKBP5*, *SGK1*, *Gal-7*
*Cell surface receptor linked signal transduction*	*INHBB*, *CAV1*	*RERG*, *GPR68*, *CXCR4*, *TGFA*, *CCBP2*, *PRLR*, *GPER*, *CCBP2*, *GEM*, *OLFM1*, *IL27RA*, *OXTR*
*Cytokine-mediated signaling pathway*		*CXCR4*, *TGFA*, *CCBP2*, *PRLR*, *CCBP2*, *IL27RA*
*G-protein coupled receptor protein signaling pathway*	*CAV1*	*RERG*, *GPR68*, *CXCR4*, *CCBP2*, *GPER*, *CCBP2*, *GEM*, *OXTR*
**Reproduction**		*STARD5*, *OXTR*
Gamete generation		*STARD5*, *OXTR*
*Female gamete generation*		*STARD5*, *OXTR*
**Metabolic process**	*HSD17B11*, *GALNT12*, *TNS3*, *TGM2*, *DIO1*, *SPINK4*, *CAV1*	*DNASE1L2*, *SERPINA3*, *NXNL2*, *EGR3*, *EGR2*, *FKBP5*, *STARD5*, *LRRFIP2*, *SGK1*, *IRX2*, *KLK5*, *HEY2*, *PPP2R5A*, *ISG20*, *ANXA8*, *SIAH2*, *C5orf4*, *NAB2*, *SOX3*, *SOX9*, *SERPINA5*, *NT5DC3*, *ALDH3B2*, *ABCA12*
Primary metabolic process	*GALNT12*, *TNS3*, *TGM2*, *SPINK4*, *CAV1*	*DNASE1L2*, *SERPINA3*, *EGR3*, *EGR2*, *FKBP5*, *STARD5*, *LRRFIP2*, *SGK1*, *IRX2*, *KLK5*, *HEY2*, *PPP2R5A*, *ISG20*, *ANXA8*, *SIAH2*, *C5orf4*, *NAB2*, *SOX3*, *SOX9*, *SERPINA5*, *NT5DC3*, *ABCA12*
*Lipid metabolic process*	*TNS3*, *CAV1*	*STARD5*, *ANXA8*, *C5orf4*, *ABCA12*
*Steroid metabolic process*		*C5orf4*, *ABCA12*
*Cholesterol metabolic process*		*C5orf4*, *ABCA12*
*Nucleobase*, *nucleoside*, *nucleotide and nucleic acid metabolic process*		*DNASE1L2*, *EGR3*, *EGR2*, *LRRFIP2*, *IRX2*, *HEY2*, *ISG20*, *NAB2*, *SOX3*, *SOX9*, *NT5DC3*
*DNA metabolic process*		*DNASE1L2*
*DNA catabolic process*		*DNASE1L2*
**System process**	*BASP1*, *SCNN1A*, *EMP1*, *GABRP*, *EYA2*	*RERG*, *FKBP5*, *OXTR*
Neurological system process	*BASP1*, *SCNN1A*, *EMP1*, *GABRP*, *EYA2*	*RERG*, *FKBP5*, *OXTR*
*Sensory perception*	*SCNN1A*, *EYA2*	*OXTR*
*Sensory perception of chemical stimulus*	*SCNN1A*	* *
*Sensory perception of taste*	*SCNN1A*	* *
**Regulation of biological process**	*SCNN1A*	* *
Regulation of vasoconstriction	*SCNN1A*	* *

## Discussion

In this paper, we investigated changes in global gene expression in response to three tamoxifen metabolites: 4OHtam, 4OHNDtam and NDtam. The main findings are that 4OHNDtam and 4OHtam strongly altered global gene expression in E_2_ treated MCF-7-cells compared to treatment with estrogen alone. The effects of 4OHNDtam and 4OHtam largely overlapped, with an overall stronger response for 4OHNDtam. NDtam had nearly no effect. We moreover identified specific genes that responded selectively to either 4OHNDtam or 4OHtam, providing new molecular insight into metabolite-specific effects with tamoxifen treatment. Our study, using physiological metabolite ratios, improves our understanding of how tamoxifen may mediate its positive and adverse effects *in vivo*.

Effects of NDtam and tamoxifen have previously been studied in MCF-7 cells. Reddel and Sutherland [30], using much higher concentrations than those observed in man (7.5–10 μM), observed that NDtam was much more potent than tamoxifen in inhibiting growth. This inhibition of cell proliferation was only partially reversed by E_2_ or not reversed at all. The results may be due to direct toxic effects of NDtam and not promoted via the influence on the ER. Hawse *et al* examined effects of 4OHtam, 4OHNDtam and NDtam in MCF-7cells in an extensive study, however they did not report on results when NDtam was used as a single drug [15]. Even with high concentrations of NDtam, mimicking that observed in man during steady state treatment, our microarray analysis clearly demonstrated that NDtam had little impact on global gene expression in E_2_-treated MCF-7 cells. This indicates that 4OHtam and 4OHNDtam are the main contributors to the anti-estrogenic effects of tamoxifen. However, NDtam, as the major tamoxifen metabolite, may still contribute to toxic effects such as crystalline retinal deposits, macular edema, and corneal changes that have been observed previously [31, 32].

In line with others [15, 33], we observed that treatment with 4OHtam and 4OHNDtam resulted in differential gene expression in E_2_ treated MCF-7 cells when compared to MCF-7 cells treated with E_2_ alone. A clear shift in gene expression was seen in the CA (Fig 1) after the treatment with 4OHtam and 4OHNDtam, however the shift was greater for 4OHNDtam than with 4OHtam. The CA showed a shift in gene expression in the same direction for 4OHtam and 4OHNDtam. When comparing the GO of the two metabolite effects separately (Fig 6), we found that genes in categories related to cancer processes such as intracellular signaling cascade were significantly down-regulated. Categories such as cell adhesion were up-regulated by both metabolites, whereas genes involved in cell cycle were down-regulated. In addition, genes in the cell motion category were down-regulated by 4OHNDtam, however not significantly by 4OHtam. Further, we found an up-regulating effect on genes related to antigen processing and presentation, a pathway associated with the emerging hallmark of cancer [34]: evasion of immune surveillance. This up-regulation was observed after treatment with 4OHNDtam, but not with 4OHtam. It is believed that cancer cells that are weakly immunogenic, and therefore harder to identify by the immune system, are the cells which eventually will form a solid tumor [34]. There are several theories surrounding how the weakly immunogenic cancer cells are able to avoid being recognized by the immune system, and how the cells became weakly immunogenic. For breast cancer cells this may involve down-regulation of components of the major histocompatibility complex [35] or, as recently proposed, by releasing proteins associated with MHC-I through exosomes [36]. Our results show an up-regulation of genes associated with antigen processing and presentation of peptide or polysaccharide antigens via MHC class II. This might represent an effect of 4OHNDtam that counteracts the evasion of immune-surveillance. However, further studies are needed to explore this hypothesis.

The differential expression observed was generally stronger during 4OHNDtam treatment. These results are in line with earlier studies suggesting that 4OHNDtam is the main compound promoting the clinical effects of tamoxifen treatment [2, 37]. We further studied the stronger regulation observed by treatment with 4OHNDtam by selecting genes that were regulated by both metabolites, but more strongly by 4OHNDtam. In the PANTHER analysis performed on these genes (S1 Fig, Table 3), the apoptosis category was of particular interest. There was an over-representation of both up-regulated genes and down-regulated genes in this category. On closer inspection the up-regulated genes (*TNS3*, *EMP1*) were positive regulators of apoptosis [38] and 4 out of 5 down-regulated genes (*CXCR4*, *Gal-7*, *FKBP5*, *SGK1)* were negative regulators of apoptosis [39–42].

An interesting finding is the strong down-regulation by 4OHNDtam of genes expressing the different keratin 6 isoforms (*KRT6a*, *KRT6b and KRT6c)*. *KRT6a* was the most down-regulated gene in our analysis (9.1–fold), and the effect on this gene was weaker after treatment with 4OHtam (1.8-fold decrease). The two-step validation by removal of the ligand (E2) (estrogen deprivation) (Fig 4) and knockdown of an important ER-coactivator (SRC-3/AIB1) (Fig 5) also suggest that the KRT6s are positively regulated by ER. It should be noted that SRC-3/AIB1 is overexpressed in 31–64% of human breast tumors [43, 44], and has been shown to increase the agonist properties of tamoxifen [45].

The keratins, also called cytokeratins (CKs), belong to the intermediate filament proteins that create an insoluble dense meshwork through the cytoplasm giving structural support to the epithelial cell. Recently, however, it was shown that CKs play a more active role in various internal cellular survival processes (e.g. proliferation and apoptosis). These proteins may undergo phosphorylation and are also part of the bridging contact between the epithelial cell and its microenvironment [46]. CK6a is the dominant isoform in the mammary gland [47] and is up-regulated in the proliferative basal cells in healing wound edges of the skin, indicating that this CK is involved in cellular proliferation and migration [48]. Knockout of CK6 is associated with reduced proliferation in the murine mammary epithelium [47]. These genes are highly up-regulated in the basaloid molecular subtype of breast cancer demonstrated by Perou and Sorlie [49]. Interestingly, co-expression of CK 5/6 in ER positive breast cancer tumors seems to define a subset of patients with a more adverse prognosis [50, 51]. Therefore, the down-regulation of CK6 by 4OHNDtam should be further explored in appropriate designed `bench-to-bed`studies since it may represent a new insight in understanding of the anti-cancer action of this active tamoxifen metabolite in ER positive breast tumors.

The present study has limitations. The standard tamoxifen metabolites used were not pure z-isomers. In the clinical situation both isomers are present although the z-isomers dominate [52]. Furthermore some isomerization may occur in the cultures during the study. Accordingly, Katzenellenbogen *et al* observed a facile geometric isomerization of anti-estrogens which happened in tissue cultures as well as cell free medium [53]. They found that the MCF-7 cells mainly accumulated the trans-isomer and at the nuclear ER mainly the trans-isomer was located. Further limitations of the study are that we studied the effects of the metabolites only at one given concentration. It must also be taken into account that the concentration of 4OHNDtam was 10 times higher than that of 4OHtam in order to mimic the clinical situation, so the stronger regulation seen by 4OHNDtam might be a reflection of the concentration differences.

## Conclusions

Conclusively, the global gene expression changes caused by 4OHNDtam treatment of estrogen treated MCF-7 cells are stronger than those of 4OHtam when using concentrations that mimic the clinical situation. NDtam caused only minimal effects. Genes encoding CytoKeratin 6 were highly down-regulated by 4OHNDtam, as well as after E2 deprivation and knockdown of SRC-3/AIB1, indicating an estrogen receptor-dependent regulation. Further studies are warranted to elucidate possible clinical applications of this finding.

## Supporting Information

S1 Fig Functional categorization of genes differentially expressed genes after 4OHtam treatment and more differentially expressed after 4OHNDtam treatment. PANTHER was used to search for over-represented categories in the ontology class Biological Process. To search for genes that were differentially expressed after treatment with 4OHNDtam and more differentially expressed after treatment with 4OHtam two subsequent rank product analyses were performed. First a rank product analysis for 4OHNDtam vs 4OHtam followed by a rank product analysis between the latter and “E2 vs 4OHtam”. Genes with rank product q-val ≤ 0.2 were selected from the rank product analysis. A p-value ≤ 0.05 was used as inclusion criterion for categories. The numbers inside the table are percentage values of the numbers above the columns. E.g. 11% of 19911 genes in the reference column can be found within the developmental process. The color intensity scales are based on the statistical significance (-log p-value) of over- and under-represented PANTHER functional categories. Red illustrates “over-represented category” where more genes than expected were found in a specific category. Blue color illustrates “under-represented category” where less genes than expected were found. Ref, Reference genes. Arrow up, up-regulated genes. Arrow down, down-regulated genes.
(TIF)

S1 Table Primers used in RT-PCR (DOC)

S2 Table Genes with increased expression after treatment with 4OHNDtam relative to E2 treatment in MCF-7 cells. (DOC)

S3 Table Genes with decreased expression after treatment with 4OHNDtam relative to E2 treatment in MCF-7 cells. (DOC)

S4 Table Genes with decreased expression after treatment with 4OHtam relative to E2 treatment in MCF-7 cells. (DOC)

S5 Table Genes with increased expression after treatment with 4OHtam relative to E2 treatment in MCF-7 cells. (DOC)

S6 Table Gene Ontology analysis J-Express 2012 (DOCX)

S7 Table Genes with decreased expression in 4OHtam and more decreased expression in 4OHNDtam (DOC)

S8 Table Genes with increased expression in 4OHtam and more increased expression in 4OHNDtam (DOC)
