# Supplementary figures and images for: The Active Tamoxifen Metabolite Endoxifen (4OHNDtam) Strongly Down-Regulates Cytokeratin 6 (CK6) in MCF-7 Breast Cancer Cells
Source: PLoS One. 2015 Apr 13;10(4):e0122339. doi: 10.1371/journal.pone.0122339 (PMC4395096; doi:10.1371/journal.pone.0122339)

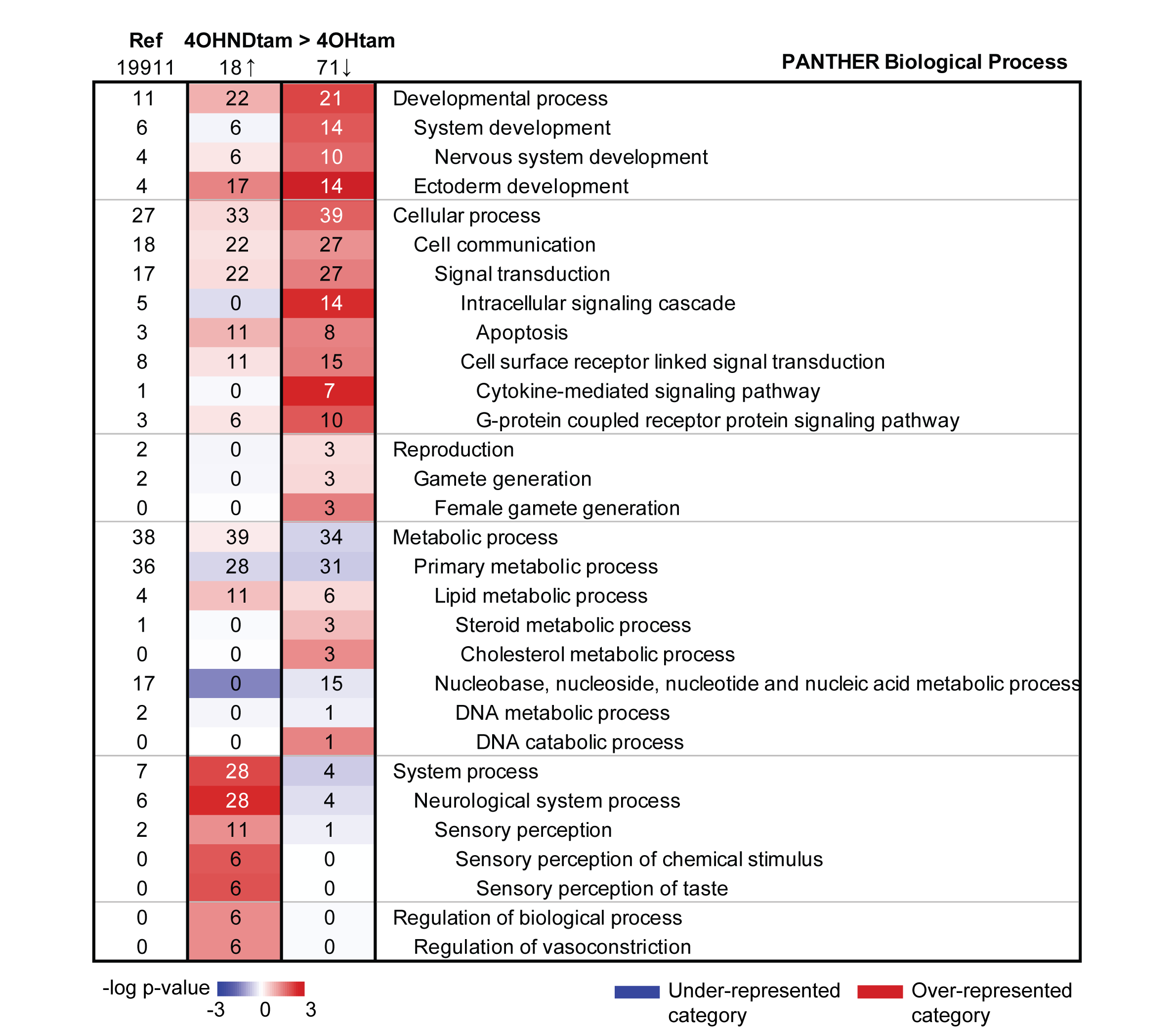

Supplement: S1 Fig — PANTHER was used to search for over-represented categories in the ontology class Biological Process. To search for genes that were differentially expressed after treatment with 4OHNDtam and more differentially expressed after treatment with 4OHtam two subsequent rank product analyses were performed. First a rank product analysis for 4OHNDtam vs 4OHtam followed by a rank product analysis between the latter and “E2 vs 4OHtam”. Genes with rank product q-val ≤ 0.2 were selected from the rank product analysis. A p-value ≤ 0.05 was used as inclusion criterion for categories. The numbers inside the table are percentage values of the numbers above the columns. E.g. 11% of 19911 genes in the reference column can be found within the developmental process. The color intensity scales are based on the statistical significance (-log p-value) of over- and under-represented PANTHER functional categories. Red illustrates “over-represented category” where more genes than expected were found in a specific category. Blue color illustrates “under-represented category” where less genes than expected were found. Ref, Reference genes. Arrow up, up-regulated genes. Arrow down, down-regulated genes. (TIF) [file pone.0122339.s001.tif]
