# Supplementary material for: The Active Tamoxifen Metabolite Endoxifen (4OHNDtam) Strongly Down-Regulates Cytokeratin 6 (CK6) in MCF-7 Breast Cancer Cells
Source: PLoS One. 2015 Apr 13;10(4):e0122339. doi: 10.1371/journal.pone.0122339 (PMC4395096; doi:10.1371/journal.pone.0122339)
Supplement: S1 Table — (DOC) [file pone.0122339.s002.doc]

**Table S1.** Primers used in RT-PCR

| **Gene** | **GeneBank ID** |  | **PCR primers (5'-3')** | **Amplicon size(bp)** | **Probe (UPL)** | **Reference Gene** |
| --- | --- | --- | --- | --- | --- | --- |
| *KRT6A* | NM_005554.3 | F | AGTTTGCCTCCTTCATCGAC | 77 | 1 | TBP |
|  |  | R | CAGCAGGGTCCACTTTGTTT |  |  |  |
| *KRT6C* | NM_173086.4 | F | GCAGTTCCACCATCAAGTACAC | 78 | 63 | TBP |
|  |  | R | AGCTGGAGGCAGCACTTTAG |  |  |  |
| *IRX2 tv2* | NM_001134222.1 | F | CGCTCGAGTCCACTACC | 119 | 70 | G6PD |
|  |  | R | CTGTGCTCGGCCCTTCTAT |  |  |  |
| *IRX3* | NM_024336.2 | F | AAAAGTTACTCAAGACAGCCTTTCCA | 92 | 57 | G6PD |
|  |  | R | GGATGAGGAGAGAGCCGATA |  |  |  |
| *IRX5* | NM_005853.5 | F | GACCTGGAGAAGAACGACGA | 92 | 45 | G6PD |
|  |  | R | GCCTTCTGCTCAGCTCCTC |  |  |  |
| *CXCR4 TV2* | NM_003467.2 | F | ATTGGGATCAGCATCGACTC | 61 | 79 | TBP |
|  |  | R | CAAACTCACACCCTTGCTTG |  |  |  |
| *COL3A1* | NM_000090.3 | F | CTGGACCCCAGGGTCTTC | 75 | 20 | PPIA |
|  |  | R | CATCTGATCCAGGGTTTCCA |  |  |  |
| *SERPINA3* | NM_001085.4 | F | TTTGGAATCCACCAGCTACA | 76 | 24 | TBP |
|  |  | R | CAGAGCCAGGAGAGGTAACATT |  |  |  |
| *CTGF* | NM_001901.2 | F | CTCCTGCAGGCTAGAGAAGC | 91 | 85 | TBP |
|  |  | R | GATGCACTTTTTGCCCTTCTT |  |  |  |
| *GPER TV4* | NM_001098201.1 | F | TAGCCCTGCTCAGGCATT | 145 | 19 | TBP |
|  |  | R | TGCTCACTCTCTGGGTACCTG |  |  |  |
