# Supplementary material for: The Active Tamoxifen Metabolite Endoxifen (4OHNDtam) Strongly Down-Regulates Cytokeratin 6 (CK6) in MCF-7 Breast Cancer Cells
Source: PLoS One. 2015 Apr 13;10(4):e0122339. doi: 10.1371/journal.pone.0122339 (PMC4395096; doi:10.1371/journal.pone.0122339)
Supplement: S2 Table — (DOC) [file pone.0122339.s003.doc]

**Table S2. Genes with increased expression after treatment with 4OHNDtam relative to E2 treatment in MCF-7 cells.**

|  |  | **Signal intesity** | | **Fold change** |
| --- | --- | --- | --- | --- |
| **SYMBOL** | **Definition** | **E2** | **4OHNDtam** | **4OHNDtam** |
| *SPINK4* | serine peptidase inhibitor, Kazal type 4 | 452 | 1534 | 3.545 |
| *TGM2* | transglutaminase 2, transcript variant 1 | 200 | 676 | 3.303 |
| *KRT4* | keratin 4 | 237 | 683 | 2.8 |
| *COL3A1* | collagen, type III, alpha 1 | 623 | 1594 | 2.536 |
| *UPK1A* | uroplakin 1A | 965 | 2428 | 2.509 |
| *HLA-DRA* | major histocompatibility complex, class II, DR alpha | 398 | 961 | 2.417 |
| *CTGF* | connective tissue growth factor | 440 | 985 | 2.332 |
| *UPK3B* | uroplakin 3B, transcript variant 2 | 508 | 1102 | 2.223 |
| *VGLL1* | vestigial like 1 (Drosophila) | 308 | 686 | 2.222 |
| *UPK2* | uroplakin 2 | 456 | 1017 | 2.188 |
| *FGD3* | FYVE, RhoGEF and PH domain containing 3, transcript variant 2 | 944 | 1974 | 2.13 |
| *DEFB1* | defensin, beta 1 | 315 | 706 | 2.127 |
| *OLFML3* | olfactomedin-like 3 | 489 | 1084 | 2.088 |
| *GALNT12* | UDP-N-acetyl-alpha-D-galactosamine:polypeptide N-acetylgalactosaminyltransferase 12 | 286 | 601 | 2.029 |
| *UPK3B* | uroplakin 3B, transcript variant 1 | 688 | 1390 | 2.008 |

Genes in table have fold change ≥ 2 and q-value = 0. FC, fold change (based on log-transformed data); q-val, q-value.
