# Supplementary material for: The Active Tamoxifen Metabolite Endoxifen (4OHNDtam) Strongly Down-Regulates Cytokeratin 6 (CK6) in MCF-7 Breast Cancer Cells
Source: PLoS One. 2015 Apr 13;10(4):e0122339. doi: 10.1371/journal.pone.0122339 (PMC4395096; doi:10.1371/journal.pone.0122339)
Supplement: S3 Table — (DOC) [file pone.0122339.s004.doc]

**Table S3.** Genes with decreased expression after treatment with 4OHNDtam relative to E2 treatment in MCF-7 cells.

|  |  | **Signal intesity** | | **Fold change** |
| --- | --- | --- | --- | --- |
| **SYMBOL** | **Definition** | **E2** | **4OHNDtam** | **4OHNDtam vs E2** |
| *KRT6A* | keratin 6A | 3816 | 409 | -9.103 |
| *SERPINA3* | serpin peptidase inhibitor, clade A, member 3 | 22257 | 2787 | -7.725 |
| *SERPINA5* | serpin peptidase inhibitor, clade A, member 5 | 2519 | 530 | -4.744 |
| *SOX3* | SRY (sex determining region Y)-box 3 | 2899 | 691 | -4.174 |
| *PKIB* | protein kinase (cAMP-dependent, catalytic) inhibitor beta, transcript variant 3 | 4121 | 1030 | -4.106 |
| *KRT6C* | keratin 6C | 887 | 205 | -4.099 |
| *MGP* | matrix Gla protein | 19149 | 4877 | -4.033 |
| *CDSN* | corneodesmosin | 1121 | 281 | -3.855 |
| *GPER* | G protein-coupled estrogen receptor 1, transcript variant 3 | 1831 | 491 | -3.826 |
| *GREB1* | GREB1 protein, transcript variant a | 3671 | 1086 | -3.565 |
| *C5orf4* | chromosome 5 open reading frame 4, transcript variant 2 | 1550 | 466 | -3.454 |
| *KRT6B* | keratin 6B | 618 | 184 | -3.301 |
| *PRSS23* | protease, serine, 23 | 4751 | 1593 | -3.104 |
| *OLFM1* | olfactomedin 1, transcript variant 1 | 4684 | 1561 | -3.018 |
| *PDZK1* | PDZ domain containing 1 | 2848 | 850 | -3.015 |
| *SGK* | serum/glucocorticoid regulated kinase | 2400 | 864 | -2.879 |
| *KLK5* | kallikrein-related peptidase 5, transcript variant 1 | 1167 | 411 | -2.857 |
| *S100A8* | S100 calcium binding protein A8 | 973 | 329 | -2.683 |
| *LRRFIP2* | leucine rich repeat (in FLII) interacting protein 2, transcript variant 2 | 2051 | 790 | -2.67 |
| *LOC653499* | similar to Galectin-7 (Gal-7) (HKL-14) (PI7) (p53-induced protein 1) | 521 | 201 | -2.636 |
| *DNASE1L2* | deoxyribonuclease I-like 2 | 751 | 275 | -2.593 |
| *SPOCK1* | sparc/osteonectin, cwcv and kazal-like domains proteoglycan (testican) 1 | 1096 | 434 | -2.556 |
| *PPP2R5A* | protein phosphatase 2, regulatory subunit B', alpha isoform | 2778 | 1099 | -2.541 |
| *KLK5* | kallikrein-related peptidase 5, transcript variant 2 | 747 | 297 | -2.529 |
| *ISG20* | interferon stimulated exonuclease gene 20kDa | 3327 | 1396 | -2.442 |
| *SLC25A18* | solute carrier family 25 (mitochondrial carrier), member 18 | 450 | 190 | -2.438 |
| *LOC728910* | PREDICTED: similar to Galectin-7 (Gal-7) (p53-induced protein 1) | 565 | 233 | -2.385 |
| *KRT16* | keratin 16 (focal non-epidermolytic palmoplantar keratoderma) | 811 | 347 | -2.38 |
| *MT2A* | metallothionein 2A | 3788 | 1544 | -2.367 |
| *ABCA12* | ATP-binding cassette, sub-family A (ABC1), member 12, transcript variant 2 | 1234 | 563 | -2.359 |
| *HIGD1A* | HIG1 domain family, member 1A, transcript variant 1 | 9126 | 3863 | -2.335 |
| *LONRF2* | LON peptidase N-terminal domain and ring finger 2 | 1085 | 441 | -2.32 |
| *RERG* | RAS-like, estrogen-regulated, growth inhibitor | 1721 | 773 | -2.301 |
| *IRX2* | iroquois homeobox 2 | 2870 | 1244 | -2.296 |
| *RARRES2* | retinoic acid receptor responder (tazarotene induced) 2 | 714 | 309 | -2.247 |
| *ANXA8* | annexin A8 | 794 | 359 | -2.226 |
| *NUPR1* | nuclear protein 1, transcript variant 1 | 740 | 325 | -2.225 |
| *CXCL12* | chemokine (C-X-C motif) ligand 12, transcript variant 1 | 2336 | 1110 | -2.158 |
| *EGR3* | early growth response 3 | 540 | 233 | -2.151 |
| *SUSD2* | sushi domain containing 2 | 946 | 432 | -2.151 |
| *CXCL12* | chemokine (C-X-C motif) ligand 12  , transcript variant 2 | 891 | 412 | -2.14 |
| *S100A9* | S100 calcium binding protein A9 (calgranulin B) | 1313 | 600 | -2.117 |
| *SUSD3* | sushi domain containing 3 | 1252 | 631 | -2.098 |
| *DMKN* | dermokine, transcript variant 1 | 1878 | 873 | -2.094 |
| *KRT17* | keratin 17 | 484 | 228 | -2.094 |
| *P8* | p8 protein (candidate of metastasis 1) (P8) | 730 | 300 | -2.091 |
| *WISP2* | WNT1 inducible signaling pathway protein 2 | 2165 | 1090 | -2.087 |
| *LOC400578* | PREDICTED: similar to Keratin, type I cytoskeletal 14 (Cytokeratin-14) | 576 | 269 | -2.085 |
| *TGFA* | transforming growth factor, alpha | 682 | 315 | -2.075 |
| *LGALS7* | lectin, galactoside-binding, soluble, 7 (galectin 7) | 401 | 188 | -2.064 |
| *MGC102966* | PREDICTED: similar to Keratin, type I cytoskeletal 16 (Cytokeratin-16) | 644 | 311 | -2.05 |
| *CCBP2* | chemokine binding protein 2 | 720 | 347 | -2.047 |
| *IFT122* | intraflagellar transport 122 homolog (Chlamydomonas) | 1194 | 570 | -2.043 |
| *RAB31* | RAB31, member RAS oncogene family | 11214 | 5614 | -2.033 |
| *C14orf132* | chromosome 14 open reading frame 132 | 771 | 365 | -2.026 |
| *OXTR* | oxytocin receptor | 1436 | 715 | -2.013 |
| *DMKN* | dermokine, transcript variant 2 | 1595 | 831 | -2.008 |
| *CXCR4* | chemokine (C-X-C motif) receptor 4, transcript variant 2 | 616 | 324 | -2.002 |

Genes in table have fold change ≥ 2 and q-value = 0.
