# Supplementary material for: The Active Tamoxifen Metabolite Endoxifen (4OHNDtam) Strongly Down-Regulates Cytokeratin 6 (CK6) in MCF-7 Breast Cancer Cells
Source: PLoS One. 2015 Apr 13;10(4):e0122339. doi: 10.1371/journal.pone.0122339 (PMC4395096; doi:10.1371/journal.pone.0122339)
Supplement: S4 Table — (DOC) [file pone.0122339.s005.doc]

**Table S4.** Genes with decreased expression after treatment with 4OHtam relative to E2 treatment in MCF-7 cells.

|  |  | **Signal intesity** | | **Fold change** |
| --- | --- | --- | --- | --- |
| **SYMBOL** | **Definition** | **E2** | **4OHtam** | **4OHtam vs E2** |
| *SERPINA3* | serpin peptidase inhibitor, clade A, member 3 | 22257 | 6639 | -3.403 |
| *GPER* | G protein-coupled estrogen receptor 1, transcript variant 3 | 1831 | 710 | -2.671 |
| *SERPINA5* | serpin peptidase inhibitor, clade A, member 5 | 2519 | 1029 | -2.452 |
| *CDSN* | corneodesmosin | 1121 | 461 | -2.448 |
| *MGP* | matrix Gla protein | 19149 | 7887 | -2.428 |
| *PDZK1* | PDZ domain containing 1 | 2848 | 1219 | -2.208 |
| *PKIB* | protein kinase (cAMP-dependent, catalytic) inhibitor beta, transcript variant 3 | 4121 | 2025 | -2.131 |

Genes in table have fold change ≥ 2 and q-value = 0.
