# Supplementary material for: The Active Tamoxifen Metabolite Endoxifen (4OHNDtam) Strongly Down-Regulates Cytokeratin 6 (CK6) in MCF-7 Breast Cancer Cells
Source: PLoS One. 2015 Apr 13;10(4):e0122339. doi: 10.1371/journal.pone.0122339 (PMC4395096; doi:10.1371/journal.pone.0122339)
Supplement: S5 Table — (DOC) [file pone.0122339.s006.doc]

**Table S5.** Genes with increased expression after treatment with 4OHtam relative to E2 treatment in MCF-7 cells.

|  |  | **Signal intensity** | | **Fold change** |
| --- | --- | --- | --- | --- |
| **SYMBOL** | **Definition** | **E2** | **4OHtam** | **4OHtam vs E2** |
| *SPINK4* | serine peptidase inhibitor, Kazal type 4 | 452 | 1527 | 3.434 |
| *COL3A1* | collagen, type III, alpha 1 | 623 | 1512 | 2.407 |
| *DEFB1* | defensin, beta 1 | 315 | 764 | 2.213 |
| *OLFML3* | olfactomedin-like 3 | 489 | 1126 | 2.188 |
| *AKR1B10* | aldo-keto reductase family 1, member B10 (aldose reductase) | 664 | 1494 | 2.152 |
| *UGT1A6* | UDP glucuronosyltransferase 1 family, polypeptide A6, transcript variant 1 | 942 | 2130 | 2.137 |
| *UGT1A6* | UDP glucuronosyltransferase 1 family, polypeptide A6, transcript variant 2 | 1230 | 2551 | 2.075 |

Genes in table have fold change ≥ 2 and q-value = 0.
