# Supplementary material for: The Active Tamoxifen Metabolite Endoxifen (4OHNDtam) Strongly Down-Regulates Cytokeratin 6 (CK6) in MCF-7 Breast Cancer Cells
Source: PLoS One. 2015 Apr 13;10(4):e0122339. doi: 10.1371/journal.pone.0122339 (PMC4395096; doi:10.1371/journal.pone.0122339)
Supplement: S6 Table — (DOCX) [file pone.0122339.s007.docx]

**Table S6. Gene Ontology analysis J-Express 2012**

|  |  |  |  |  |
| --- | --- | --- | --- | --- |
| ***4OHNDtam up-regulated genes*** |  |  |  |  |
| **Gene Ontology term** | **Exact test p-value** | **Genes in selection** | **Genes in reference** | **Enrichment** |
| lipid metabolic process | 2.00E-03 | 4 | 271 | 7.35 |
| carbohydrate metabolic process | 6.00E-03 | 4 | 358 | 5.564 |
| response to carbohydrate stimulus | 9.17E-04 | 3 | 91 | 16.417 |
| antigen receptor-mediated signaling pathway | 5.23E-06 | 5 | 118 | 21.101 |
| immune response-activating cell surface receptor signaling pathway | 6.59E-06 | 5 | 124 | 20.08 |
| immune response-regulating cell surface receptor signaling pathway | 8.84E-06 | 5 | 132 | 18.863 |
| antigen processing and presentation of peptide or polysaccharide antigen via MHC class II | 2.38E-05 | 2 | 2 | 497.99 |
| antigen processing and presentation of polysaccharide antigen via MHC class II | 2.38E-05 | 2 | 2 | 497.99 |
| cell adhesion | 5.00E-03 | 6 | 785 | 3.806 |
| developmental process | 1.72E-14 | 37 | 4451 | 4.14 |
| blood vessel development | 1.66E-04 | 6 | 396 | 7.545 |
| nervous system development | 3.00E-03 | 10 | 1748 | 2.849 |
| metabolic process | 5.32E-05 | 29 | 6838 | 2.112 |
| regulation of metabolic process | 9.07E-05 | 19 | 3594 | 2.633 |
| ion transport | 4.77E-05 | 8 | 635 | 6.274 |
| signal transduction | 1.83E-08 | 26 | 3761 | 3.443 |
|  |  |  |  |  |
| ***4OHNDtam down-regulated genes*** |  |  |  |  |
| **Gene Ontology term** | **Exact test p-value** | **Genes in selection** | **Genes in reference** | **Enrichment** |
| programmed cell death | 9.14E-04 | 11 | 923 | 3.144 |
| cellular process | 1.76E-11 | 62 | 6831 | 2.394 |
| regulation of cell migration | 6.31E-06 | 7 | 174 | 10.613 |
| developmental process | 4.47E-20 | 62 | 4451 | 3.675 |
| nervous system development | 3.85E-09 | 26 | 1748 | 3.924 |
| response to stimulus | 7.29E-13 | 51 | 4492 | 2.995 |
| signaling | 4.27E-09 | 40 | 3828 | 2.757 |
| signal transduction | 8.58E-09 | 39 | 3761 | 2.735 |
|  |  |  |  |  |
| ***4OHtam up-regulated genes*** |  |  |  |  |
| **Gene Ontology term** | **Exact test p-value** | **Genes in selection** | **Genes in reference** | **Enrichment** |
| carbohydrate metabolic process | 0.007 | 3 | 358 | 7.865 |
| carbohydrate kinase activity | 2.53E-04 | 2 | 20 | 93.852 |
| carbohydrate phosphorylation | 3.55E-04 | 2 | 24 | 78.21 |
| developmental process | 3.15E-11 | 23 | 4451 | 4.85 |
| system development | 1.86E-09 | 18 | 3138 | 5.383 |
| homeostatic process | 9.90E-04 | 6 | 1075 | 5.238 |
| response to stimulus | 1.33E-08 | 20 | 4492 | 4.179 |
| signal transduction | 1.11E-06 | 16 | 3761 | 3.993 |
| signaling | 1.40E-06 | 16 | 3828 | 3.923 |
|  |  |  |  |  |
| ***4OHtam down-regulated genes*** |  |  |  |  |
| **Gene Ontology term** | **Exact test p-value** | **Genes in selection** | **Genes in reference** | **Enrichment** |
| programmed cell death | 3.42E-04 | 7 | 923 | 5.364 |
| cell death | 5.24E-04 | 7 | 992 | 4.991 |
| positive regulation of extrinsic apoptotic signaling pathway | 7.78E-04 | 2 | 27 | 52.392 |
| metabolic process | 1.22E-04 | 22 | 6838 | 2.276 |
| cellular process | 3.42E-06 | 25 | 6831 | 2.589 |
| developmental process | 7.77E-10 | 25 | 4451 | 3.973 |
| nervous system development | 9.73E-07 | 13 | 1748 | 5.26 |
| positive regulation of G-protein coupled receptor protein signaling pathway | 1.07E-04 | 2 | 9 | 157.176 |

Gene ontology analysis performed on J-Express 2012. Gene ontology terms and associations updated to latest version 6.11.2014.
