# Supplementary material for: The Active Tamoxifen Metabolite Endoxifen (4OHNDtam) Strongly Down-Regulates Cytokeratin 6 (CK6) in MCF-7 Breast Cancer Cells
Source: PLoS One. 2015 Apr 13;10(4):e0122339. doi: 10.1371/journal.pone.0122339 (PMC4395096; doi:10.1371/journal.pone.0122339)
Supplement: S7 Table — (DOC) [file pone.0122339.s008.doc]

**Table S7.** Genes with decreased expression in 4OHtam and more decreased expression in 4OHNDtam

|  |  |  | **Fold Change** | |
| --- | --- | --- | --- | --- |
| **SYMBOL** | **Definition** | **q-value (rank product)** | **4OHtam vs E2** | **4OHNDtam vs E2** |
| *KRT6A* | keratin 6A | 0.02 | -1.80 | -9.10 |
| *SERPINA3* | serpin peptidase inhibitor, clade A (alpha-1 antiproteinase, antitrypsin), member 3 | 0.00 | -3.40 | -7.73 |
| *SERPINA5* | serpin peptidase inhibitor, clade A (alpha-1 antiproteinase, antitrypsin), member 5 | 0.00 | -2.45 | -4.74 |
| *SOX3* | SRY (sex determining region Y)-box 3 | 0.00 | -1.98 | -4.17 |
| *PKIB* | protein kinase (cAMP-dependent, catalytic) inhibitor beta, transcript variant 3 | 0.01 | -2.13 | -4.11 |
| *MGP* | matrix Gla protein | 0.01 | -2.43 | -4.03 |
| *CDSN* | corneodesmosin | 0.01 | -2.45 | -3.86 |
| *GPER* | G protein-coupled estrogen receptor 1, transcript variant 3 | 0.01 | -2.67 | -3.83 |
| *GPER* | G protein-coupled estrogen receptor 1, transcript variant 3 | 0.01 | -2.56 | -3.75 |
| *C5orf4* | chromosome 5 open reading frame 4, transcript variant 2 | 0.01 | -1.28 | -3.45 |
| *MGP* | matrix Gla protein | 0.00 | -2.05 | -3.27 |
| *PRSS23* | protease, serine, 23 | 0.05 | -1.13 | -3.10 |
| *OLFM1* | olfactomedin 1, transcript variant 1 | 0.01 | -1.58 | -3.02 |
| *PDZK1* | PDZ domain containing 1 | 0.02 | -2.21 | -3.02 |
| *SGK* | serum/glucocorticoid regulated kinase | 0.05 | -1.80 | -2.88 |
| *KLK5* | kallikrein-related peptidase 5, transcript variant 1 | 0.04 | -1.86 | -2.86 |
| *LRRFIP2* | leucine rich repeat (in FLII) interacting protein 2, transcript variant 2 | 0.01 | -1.32 | -2.67 |
| *LOC653499* | similar to Galectin-7 (Gal-7) (HKL-14) (PI7) (p53-induced protein 1) | 0.03 | -1.79 | -2.64 |
| *DNASE1L2* | deoxyribonuclease I-like 2 | 0.01 | -1.52 | -2.59 |
| *SPOCK1* | sparc/osteonectin, cwcv and kazal-like domains proteoglycan (testican) 1 | 0.02 | -1.27 | -2.56 |
| *PPP2R5A* | protein phosphatase 2, regulatory subunit B', alpha isoform | 0.01 | -1.63 | -2.54 |
| *KLK5* | kallikrein-related peptidase 5, transcript variant 2 | 0.01 | -1.77 | -2.53 |
| *ISG20* | interferon stimulated exonuclease gene 20kDa | 0.05 | -1.14 | -2.44 |
| *SLC25A18* | solute carrier family 25 (mitochondrial carrier), member 18, nuclear gene encoding mitochondrial protein | 0.01 | -1.84 | -2.44 |
|  | BX109404 NCI_CGAP_Br2 cDNA clone IMAGp998G234083, mRNA sequence | 0.02 | -1.65 | -2.44 |
| *HIGD1A* | HIG1 domain family, member 1A, transcript variant 1 | 0.02 | -1.35 | -2.34 |
| *HIGD1A* | HIG1 domain family, member 1A, transcript variant 1 | 0.04 | -1.27 | -2.31 |
| *RERG* | RAS-like, estrogen-regulated, growth inhibitor | 0.04 | -1.55 | -2.30 |
| *IRX2* | iroquois homeobox 2 | 0.04 | -1.59 | -2.30 |
| *DMKN* | dermokine, transcript variant 1 | 0.04 | -1.35 | -2.09 |
| *CCBP2* | chemokine binding protein 2 | 0.04 | -1.33 | -2.05 |
| *RAB31* | RAB31, member RAS oncogene family | 0.05 | -1.22 | -2.03 |
| *DMKN* | dermokine, transcript variant 2 | 0.04 | -1.38 | -2.01 |
| *HSPA12A* | heat shock 70kDa protein 12A | 0.04 | -1.32 | -1.95 |
| *C6orf126* | chromosome 6 open reading frame 126 | 0.05 | -1.64 | -1.92 |
| *GPR68* | G protein-coupled receptor 68 | 0.05 | -1.34 | -1.78 |
| *STARD5* | StAR-related lipid transfer (START) domain containing 5 | 0.05 | -1.44 | -1.76 |

Genes in table have a rank product q-value ≤0.05.
