# Supplementary material for: The Active Tamoxifen Metabolite Endoxifen (4OHNDtam) Strongly Down-Regulates Cytokeratin 6 (CK6) in MCF-7 Breast Cancer Cells
Source: PLoS One. 2015 Apr 13;10(4):e0122339. doi: 10.1371/journal.pone.0122339 (PMC4395096; doi:10.1371/journal.pone.0122339)
Supplement: S8 Table — (DOC) [file pone.0122339.s009.doc]

**Table S8.** Genes with increased expression in 4OHtam and more increased expression in 4OHNDtam

|  |  |  | **Fold Change** | |
| --- | --- | --- | --- | --- |
| **SYMBOL** | **Definition** | **q-value (rank product)** | **4OHtam vs E2** | **4OHNDtam vs E2** |
| *TGM2* | transglutaminase 2 (C polypeptide, protein-glutamine-gamma-glutamyltransferase), transcript variant 1. | 0.033 | 1.582 | 3.303 |
| *GALNT12* | UDP-N-acetyl-alpha-D-galactosamine:polypeptide N-acetylgalactosaminyltransferase 12 | 0.033 | 1.464 | 2.029 |
| *GABRP* | gamma-aminobutyric acid A receptor, pi | 0.033 | 1.28 | 1.88 |

Genes in table have a rank product q-value ≤0.05.
